# Prophylactic Cranial Irradiation in MRI-Staged Limited-Stage Small-Cell Lung Cancer: A Systematic Review and Meta-Analysis of Survival and Neurocognitive Outcomes

**DOI:** 10.3390/cancers18121970

**Published:** 2026-06-17

**Authors:** Shaoli Li, Xin Zheng, Yinnan Meng, Gang Chen, Caining Zhao, Sijin Zhong, Huixia Li, Rui Bai, Ying Dong, Feng-Ming (Spring) Kong

**Affiliations:** 1 Department of Clinical Oncology, The University of Hong Kong-Shenzhen Hospital, Shenzhen 518000, China; 2 Department of Clinical Oncology, LKS Faculty of Medicine, The University of Hong Kong, Hong Kong, China; 3 Translational Medicine Centre, The University of Hong Kong-Shenzhen Hospital, Shenzhen 518000, China; 4 Department of Medical Oncology, The Second Affiliated Hospital, Zhejiang University School of Medicine, Hangzhou 310009, China; 5 Department of Clinical Oncology, Shenzhen Key Laboratory for Cancer Metastasis, Shenzhen 518000, China

**Keywords:** prophylactic cranial irradiation, limited-stage small-cell lung cancer, magnetic resonance imaging, survival benefit, meta-analysis

## Abstract

**Background:** The necessity of prophylactic cranial irradiation (PCI) for limited-stage small-cell lung cancer (LS-SCLC) is increasingly debated in the era of high-resolution magnetic resonance imaging (MRI). This meta-analysis evaluated the survival and neurocognitive effects of PCI in LS-SCLC patients confirmed negative for brain metastases (BM) by pre-PCI MRI. **Methods:** Following PRISMA guidelines, a systematic search of PubMed, Embase, and Cochrane Library was conducted through 23 September 2025. Pooled hazard ratios (HRs) and risk ratios (RRs) were calculated to assess overall survival (OS), progression-free survival (PFS), brain metastasis-free survival (BMFS), and BM rate. **Results:** Eleven studies involving 1894 patients were synthesized. PCI conferred a significant survival advantage, reducing the risk of death by 31% (HR = 0.69, 95% CI: 0.61–0.78), which translated to an absolute OS improvement of 8.5% at 2 years and 6.5% at 5 years. Substantial improvements were also observed in PFS (HR = 0.71, 95% CI: 0.60–0.84) and BMFS (HR = 0.53, 95% CI: 0.32–0.88), alongside a 36% reduction in BM rate (RR = 0.64, 95% CI: 0.53–0.77). Notably, PCI maintained its OS benefit even when compared directly against active MRI surveillance. While neurocognitive adverse events were reported, evidence suggests that these effects are predominantly transient and domain-specific, without a sustained decrease in global quality of life. **Conclusions:** In the MRI-staging era, PCI remains a significant therapeutic option for LS-SCLC. It offers a favorable risk–benefit profile by significantly enhancing survival while maintaining manageable neurocognitive safety.

## Simple Summary

With the widespread adoption of high-resolution magnetic resonance imaging (MRI), the traditional role of prophylactic cranial irradiation (PCI) in limited-stage small-cell lung cancer is being re-evaluated. Our study aimed to determine whether PCI still offers a survival advantage for patients with MRI-confirmed negative brain metastases following initial definitive therapy. In a large cohort of 1894 patients, we found that PCI significantly improves overall survival, reducing the risk of death by 31%, which translates to an absolute survival gain of 8.5% at 2 years. Crucially, this survival benefit was maintained even when compared directly against active MRI surveillance. While neurocognitive side effects were reported, they were predominantly transient and did not compromise global quality of life. Our findings reinforce PCI as a significant therapeutic option, providing robust evidence that imaging surveillance alone cannot replace the consolidative benefits of preventative radiation.

## 1. Introduction

Lung cancer remains the primary cause of cancer-related mortality globally [1]. Among its subtypes, small-cell lung cancer (SCLC) is particularly aggressive, accounting for roughly 13–15% of cases [2,3]. A defining clinical hallmark of SCLC is its high propensity for early brain metastases (BMs), which occur in up to 50% of patients and profoundly impair both quality of life and overall survival (OS) [4,5]. For limited-stage SCLC (LS-SCLC), current clinical guidelines, including those from the NCCN, recommend prophylactic cranial irradiation (PCI) for patients achieving a favorable response to initial therapy [6,7]. This was established by landmark meta-analyses in the 1990s, which suggested significant gains in both intracranial control and OS [8,9,10,11].

However, the diagnostic framework for brain staging has been fundamentally reshaped. The foundational evidence supporting PCI was established in an era when computed tomography (CT) or even no imaging was the standard. The widespread use of brain magnetic resonance imaging (MRI) for detecting BM has prompted a re-evaluation of the role of PCI in patients with LS-SCLC [12]. Compared to CT, contrast-enhanced magnetic resonance imaging (MRI) offers significantly higher sensitivity and spatial resolution, capable of detecting millimeter-sized asymptomatic lesions. This shift to “negative MRI staging” has introduced a “stage migration” effect: many patients previously classified as “negative” by CT would now be identified as BM-positive by MRI. Therefore, in historical cohorts, PCI may have functioned as an early therapeutic intervention for occult, pre-existing metastases rather than a truly prophylactic measure [13]. For a patient who is confirmed a “true negative” by modern high-resolution MRI following initial chemoradiotherapy, the absolute survival gain of consolidative radiation becomes a subject of intense debate. This clinical dilemma is further intensified by the growing emphasis on long-term quality of life. While PCI effectively reduces the rate of BM, it is associated with well-documented treatment-induced neurotoxicity, including a decline in memory, executive function, and global cognitive health [14]. Moreover, evidence from extensive-stage SCLC trials has already demonstrated no OS advantage for PCI compared with active MRI surveillance [15], though the biology and prognostic drivers differ considerably between LS-SCLC and extensive-stage SCLC. These findings have prompted a shift toward a more nuanced, risk-adapted approach in clinical practice [6].

In the contemporary MRI era, evidence regarding the value of PCI remains conflicting. The controversy is most acute in patients with confirmed negative pre-PCI MRI, where the survival benefit of prophylaxis versus regular MRI surveillance remains poorly defined. Several studies, including a recent meta-analysis, suggest that PCI improves survival outcomes among patients who have undergone baseline MRI [11,16,17]. Supporting this view, a previous study reported that for LS-SCLC patients without BM on MRI after definitive chemoradiotherapy, PCI was associated with a reduced BM rate and improved OS [18]. Conversely, other studies suggested that PCI did not significantly improve OS and was associated with notable neurotoxicity [19,20]. Another study reported that while PCI reduced the risk of BM, it did not significantly prolong OS compared with active MRI surveillance [21]. This lack of consensus underscores a critical gap in clinical evidence: the necessity of PCI, specifically for LS-SCLC patients who are confirmed negative for BM by high-resolution MRI after completing initial definitive therapy, remains poorly defined.

This meta-analysis aimed to evaluate the efficacy of PCI specifically in cases of LS-SCLC confirmed negative for BM by MRI following initial treatment, and clarify whether PCI offers a survival advantage over active MRI surveillance in the contemporary imaging era. Furthermore, a secondary objective was to synthesize available data on neurocognitive outcomes to provide a comprehensive, evidence-based risk–benefit assessment for clinical decision-making.

## 2. Methods

In accordance with the Preferred Reporting Items for Systematic Reviews and Meta-analyses (PRISMA) guidelines (see Supplementary Table S1) [22], two authors (SLL and XZ) independently carried out a search of the literature, quality assessment, and data extraction processes for this meta-analysis (PROSPERO ID: CRD420251163608).

### 2.1. Search Strategy and Study Selection

A systematic search was conducted for all possibly peer-reviewed articles using PubMed, Embase, and the Cochrane Library from database inception until 23 September 2025. The search used the following keywords: “small cell lung carcinoma”, “small cell lung cancer”, “prophylactic brain irradiation”, “prophylactic cranial irradiation”, “PCI”, and “whole-brain radiotherapy”.

The inclusion criteria were as follows: (1) studies involving patients with LS-SCLC; (2) studies involving patients without BM, as confirmed by brain MRI after initial treatment; and (3) studies comparing outcomes between PCI and non-PCI groups, including OS, progression-free survival (PFS), rate of BM, or brain metastasis-free survival (BMFS). The exclusion criteria were as follows: (1) non-original articles, including abstracts, reviews, letters, comments, and case reports; (2) studies with insufficient data for extraction; and (3) non-English publications.

### 2.2. Data Extraction and Quality Evaluation

The following data from the included studies were extracted: general characteristics (including the first author, publication year, sample size, and study design), patients’ information (including age, gender, smoking history, tumor stage, previous treatment, response to the primary treatment, and PCI strategy), and study outcomes. The primary outcome for this meta-analysis was OS, while secondary outcomes included PFS, the rate of BM, BMFS, and neurocognitive effects. The quality of the included studies was evaluated using the Newcastle–Ottawa Scale (NOS). NOS is an eight-item scale covering three domains: selection of study groups, comparability of the groups, and ascertainment of exposure or outcome (for case–control or cohort studies, respectively). The maximum possible score on the NOS is nine points, with higher scores indicating better quality [23]. Studies with a score of 7 or higher were considered high quality. Any discrepancies were resolved through discussion and consensus among all authors.

### 2.3. Statistical Analysis

The primary endpoint of this study was OS, defined as the time from randomization to death from any cause. Key secondary endpoints were PFS (time to disease progression or death), BM (rate of confirmed brain lesions by MRI), BMFS (time to brain metastasis or death), and neurocognitive effect.

Statistical analyses were primarily performed using R software (version 4.5.1, R Foundation for Statistical Computing, Vienna, Austria) for the core meta-analysis, subgroup comparisons, meta-regression, and assessment of publication bias via Egger’s and Begg’s tests. Additionally, Stata (version 14.0, Stata Corp, College Station, TX, the United States of America) was specifically employed for sensitivity analyses to ensure the robustness of our findings. For OS, PFS, and BMFS, a hazard ratio (HR) with a 95% confidence interval (CI) was extracted from the included studies. When direct data were unavailable, Engauge Digitizer software (version 12.1) was used to extract relevant data from Kaplan–Meier (K-M) survival curves, and an HR with 95% CI was estimated using the method described in a previous study [24]. For studies reporting the rate of BM, the risk ratio (RR) was pooled. The I^2^ was used to evaluate the heterogeneity among the included studies. A random-effects model was applied when substantial heterogeneity was present (I^2^ > 50%). Otherwise, a common-effect model was utilized. Subgroup analyses were performed based on study design, initial treatment, and MRI examination protocol. Robustness of the pooled results was assessed through a sensitivity analysis, which involved systematically excluding each study in turn. Funnel plots, Egger’s test, and Begg’s test were employed to evaluate publication bias. All *p*-values were two-sided, and *p*  <  0.05 was considered statistically significant.

## 3. Results

### 3.1. Study Selection and Baseline Characteristics

**Figure 1:**
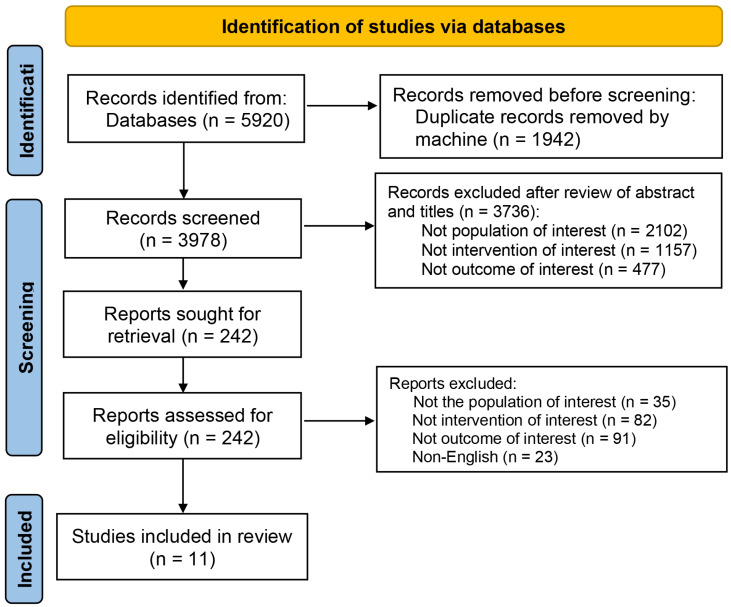
Flow diagram of the literature search (n: number of studies).

A total of 11 retrospective studies comprising 1894 patients with LS-SCLC were eligible for inclusion (see Figure 1). The detailed characteristics of the included studies are presented in Table 1. Following completion of definitive primary therapy and negative intracranial staging via MRI, 914 patients received PCI while 980 underwent observation or surveillance. Negative MRI was defined across all cohorts as the radiological absence of BM on scans performed strictly after primary treatment but before PCI enrollment. Specifically, four studies explicitly documented the use of contrast-enhanced MRI, while the remaining seven utilized MRI without specifying contrast media usage.

To mitigate potential selection bias, eight studies employed propensity score matching to achieve balanced baseline cohorts. Initial treatment modalities predominantly consisted of concurrent chemoradiotherapy (*n* = 9), with surgery or sequential radiotherapy utilized in the remaining studies. The most common PCI fractionation was 25 Gy in 10 fractions (*n* = 5), with others ranging from 24 to 30 Gy. All included studies mandated brain MRI after initial treatment/before PCI, and six studies implemented scheduled post-treatment MRI surveillance. Regarding reported outcomes, OS was reported across all 11 studies, while secondary endpoints, including PFS, MRI-confirmed BM rate, and BMFS, were reported in five studies each. Notably, the included studies did not distinguish between symptomatic and asymptomatic brain lesions in their reporting of intracranial failure. Data on neurocognitive effects were reported in only two studies.

### 3.2. Impact of PCI on OS

**Figure 2:**
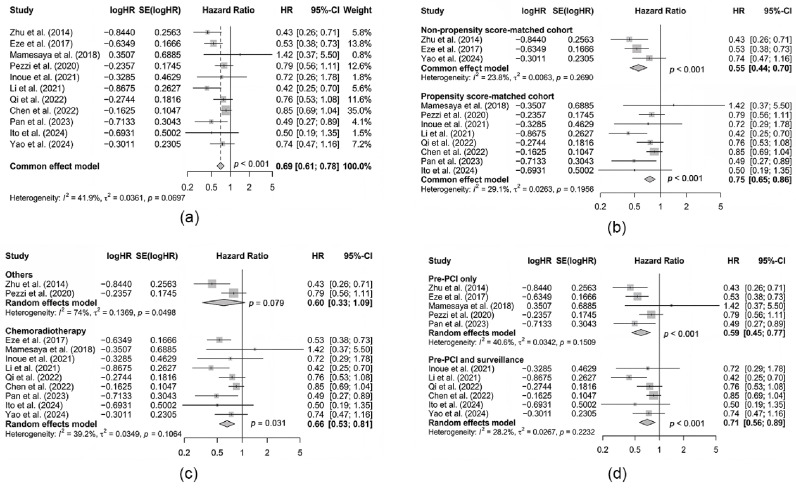
Forest plots for overall survival (OS). This figure shows significant differences in OS between groups for: (**a**) PCI vs. no PCI; (**b**) subgroup by study design; (**c**) subgroup by initial treatment; and (**d**) subgroup by MRI protocol. Reference: [11,16,18,19,21,25,26,27,28,29,30] Abbreviation: HR: hazard ratio; PCI: prophylactic cranial irradiation.

Meta-analysis of all 11 cohorts suggested that PCI was associated with a statistically significant and clinically robust improvement in OS (HR = 0.69, 95% CI: 0.61–0.78, see Figure 2a). In long-term follow-up studies, this relative benefit corresponded to a mean absolute OS improvement of 8.5% (range: 5–12%) at 2 years and 6.5% (range: 5–19%) at 5 years. Although moderate heterogeneity was observed (I^2^ = 41.9%), subsequent subgroup analyses effectively delineated potential confounding factors. Notably, the survival benefit appeared sensitive to the patient’s therapeutic response status after initial treatment. Specifically, the only trial exclusively enrolling patients with a complete response (CR) demonstrated no significant OS improvement [19], whereas studies including both CR and partial response (PR) patients showed clear therapeutic advantages. This suggests that the OS benefit of PCI may be less pronounced in the CR subgroup, potentially due to the efficacy of salvage therapies for subsequent brain metastases [31].

To further investigate the origins of heterogeneity, additional stratifications were performed based on study design, initial treatment modality, and MRI protocols. When stratified by study design, heterogeneity decreased within each subgroup (Figure 2b). Regarding treatment modalities, heterogeneity remained moderate in the chemoradiotherapy cohort but was high in patients receiving alternative treatments (Figure 2c). Furthermore, stratification by MRI protocols effectively mitigated study heterogeneity, with a marked reduction in I^2^ observed in both the pre-PCI screening subgroup (PCI vs. observation) and the combined pre-PCI plus MRI surveillance subgroup (PCI vs. regular MRI monitoring). This indicates that the rigor of intracranial surveillance was a primary source of the initial variability across studies (Figure 2d).

To quantitatively evaluate potential moderators of heterogeneity, univariable meta-regression was conducted. Analysis using publication year as a covariate revealed a trend toward an attenuated OS benefit in more recent cohorts (Estimate = 0.0461, *p* = 0.108). This observation likely reflects the “stage migration” effect, as advancements in MRI resolution have enabled more precise exclusion of patients with occult metastases, narrowing the survival gap between PCI and contemporary surveillance. The bubble plot visually corroborated this relationship, illustrating a gradual upward trend in HR estimates over time (Figure S1a). The size of each bubble represents the weighting of individual studies, with larger bubbles indicating higher precision. Meta-regression for complete response (CR) rates showed no statistical association with the OS benefit (*p* = 0.236), and the corresponding bubble plot suggested a relatively flat regression slope (Figure S1b). This suggests that the therapeutic advantage of PCI remains remarkably consistent across cohorts regardless of the proportion of patients achieving a CR.

### 3.3. Impact of PCI on PFS, BM, and BMFS

**Figure 3:**
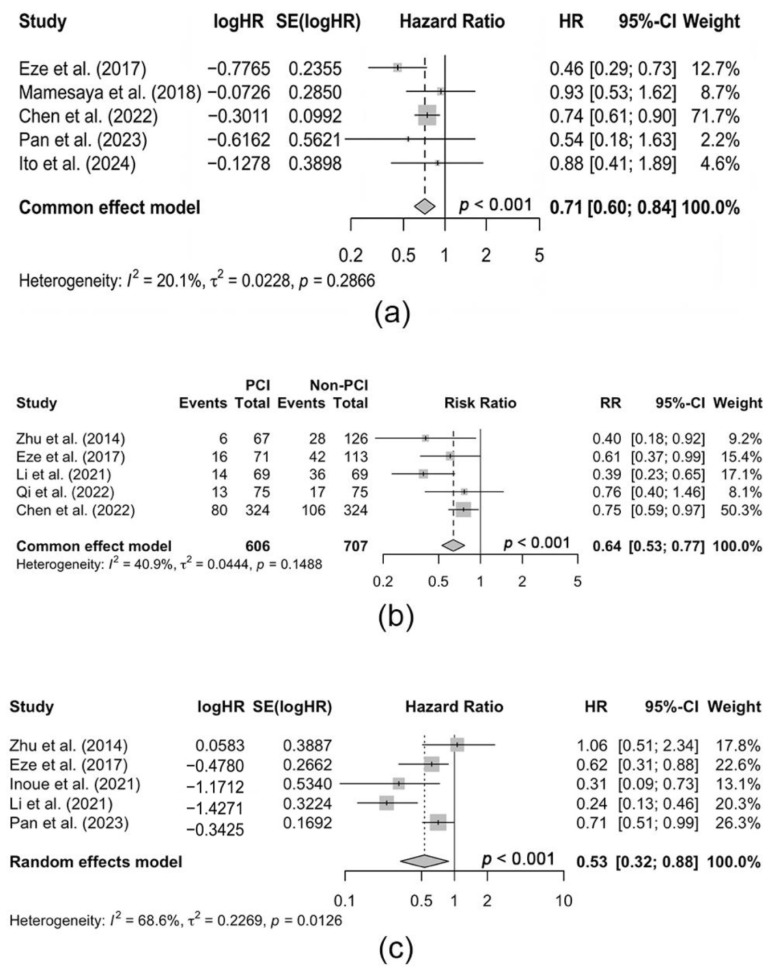
Forest plots of the pooled estimates from the meta-analysis. This figure shows significant differences between the PCI and no-PCI groups in (**a**) PFS (HR); (**b**) BM rate (RR); and (**c**) BMFS (HR). Reference: [11,16,18,19,21,25,26,28,29] Abbreviation: HR: hazard ratio; PCI: prophylactic cranial irradiation; RR: risk ratio.

Five studies compared the PFS between PCI and non-PCI groups, showing that PCI was also linked to a significant increase in PFS (HR = 0.71, 95% CI: 0.60–0.84, see Figure 3a). Additionally, the pooled RR for the rate of BM across five studies was 0.64 (95% CI: 0.53–0.77, as shown in Figure 3b), indicating that PCI substantially lowered the risk of BM compared to the non-PCI group. Importantly, the BMFS was also notably improved with PCI (HR = 0.53, 95% CI: 0.32–0.88; see Figure 3c).

### 3.4. Evaluation of Neurocognitive Effects and Quality of Life (QoL)

Among the 11 included studies, only 2 reported neurocognitive effects associated with PCI; thus, meta-analysis was not possible. One study documented neurocognitive impairment in 5 out of 32 patients (15.6%) who underwent PCI, while no such deficits were observed in the non-PCI group [28]. Another study reported one case of leukoencephalopathy following PCI in the treatment arm [26]. Taken together, these two studies suggest that PCI may be associated with neurocognitive adverse events, including impairment and leukoencephalopathy.

A broader synthesis of available randomized trial data indicates that while PCI may induce measurable neurocognitive sequelae, these effects are predominantly low-grade and transient (see Table 2) [15,32,33,34,35,36,37]. Specifically, most studies using broad screening tools (e.g., Mini-Mental State Examination and Addenbrooke’s Cognitive Examination-Revised) found no significant long-term differences in global cognitive function between groups [15,34,35,37]. Notwithstanding these global findings, subtle domain-specific deficits have been identified. For instance, a statistically significant decline in verbal recall was noted at the one-year mark in one trial [34], while another reported increased physician-assessed, low-grade memory issues [36]. Regarding patient-reported outcomes, negative impacts (e.g., on fatigue) were mainly short-term and often below thresholds for clinical significance, with no significant differences in global QoL between arms at 1 year or beyond in most studies [33,34,36,37].

### 3.5. Assessment of Study Quality, Sensitivity, and Publication Bias

Based on the NOS, all included studies clearly defined diagnostic criteria for LS-SCLC. The average score across eleven studies was 7.6, indicating that the methodological quality of the included literature was acceptable. The sections with lower scores mainly related to patient selection and the adequacy of follow-up duration for survival outcomes (as shown in Table 3).

Beyond the standard leave-one-out analysis, a restricted sensitivity analysis was performed on the six studies that implemented scheduled post-treatment MRI surveillance. In this specific subgroup, heterogeneity was markedly reduced (I^2^ = 28.2%), and the pooled survival benefit remained significant under the fixed-effects model (HR = 0.76, 95% CI: 0.65–0.88, *p* < 0.001). These findings provide a robust answer to whether PCI maintains its value in the setting of active surveillance, confirming that prophylaxis still offers a tangible survival gain over imaging alone.

**Figure 4:**
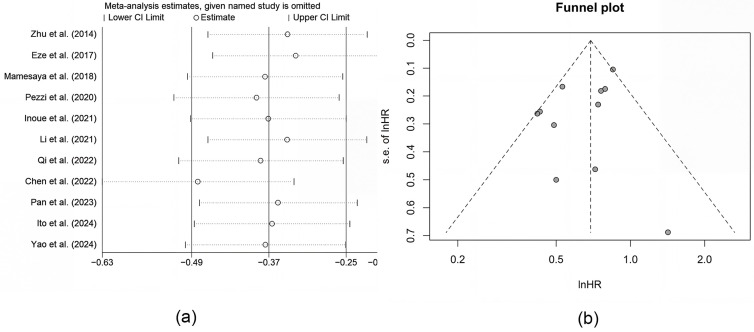
(**a**) Sensitivity analysis for OS. (**b**) Funnel plot for OS. Sensitivity analysis shows no individual study significantly altered the pooled OS. The funnel plot indicates no evident publication bias. Abbreviation: CI: confidence interval; HR: hazard ratio; s.e.: standard error. Reference: [11,16,18,19,21,25,26,27,28,29,30].

The general sensitivity analysis indicated that no single study had an undue influence on the pooled estimates (as shown in Figure 4a), which suggested the results of this meta-analysis were reliable. Furthermore, publication bias was assessed using both Egger’s and Begg’s tests, and funnel plots were generated for OS. The funnel plot appeared symmetric, and no significant publication bias was detected (see Figure 4b, Egger’s *p*  =  0.232; Begg’s *p*  = 0.484).

## 4. Discussion

This meta-analysis of 11 studies including nearly two thousand cases suggested that in patients of LS-SCLC staged with pre-PCI MRI, PCI significantly improved OS (HR = 0.69), PFS (HR = 0.71), and BMFS (HR = 0.53), while reducing the rate of BM (RR = 0.64). By strictly evaluating the MRI era, our analysis minimized the “stage migration” noise inherent in historical cohorts where outdated imaging potentially confounded truly prophylactic benefits with the early treatment of undetected, pre-existing metastases. Specifically, PCI was associated with a 31% reduction in mortality risk, translating to an absolute OS difference of 8.5% at 2 years and 6.5% at 5 years among patients with confirmed negative pre-PCI MRI.

These findings reinforce the therapeutic value of PCI in LS-SCLC, offering a necessary update to broader historical syntheses of SCLC management. While larger systematic reviews provide a comprehensive panoramic view across several decades, their reliance on a mix of imaging eras may obscure the specific efficacy of PCI in the modern diagnostic framework. The divergent outcomes between limited-stage and extensive-stage disease, where trials like the Japanese study showed no survival benefit, likely stem from distinct disease biology [15]. In extensive-stage disease, prognosis is dominated by systemic progression [38], whereas in LS-SCLC responding well to chemoradiotherapy, the brain becomes a common site of relapse due to the blood–brain barrier [39]. This explains why the consolidative role of PCI in eradicating occult micro-metastases maintains significant clinical weight in the limited-stage setting but not in extensive-stage populations [40].

By focusing on patients who underwent MRI screening after initial therapy, this analysis provides novel evidence that PCI significantly improves PFS (reducing the risk of disease progression by 29%) and substantially lowers the BM rate by 36%. These definitive outcomes address a key methodological limitation of prior studies, which largely evaluated PCI in the absence of routine cranial MRI screening [8,41,42]. The subsequent improvement in PFS is a critical mediator for preserving neurological function and quality of life. Thus, within modern management paradigms, PCI should be regarded as an intervention that secures both systemic disease control and patient-centered outcomes [43,44]. The landscape of LS-SCLC treatment is further evolving with the clinical application of the ADRIATIC trial, which established durvalumab consolidation as a new standard of care [45]. As systemic control improves through chemoimmunotherapy, the synergy between immune checkpoint inhibitors and local prophylactic radiation remains a critical area for investigation. Improved systemic control may theoretically increase the cumulative risk of brain relapses over time as patients survive longer, which could further justify the evaluation of intracranial prophylaxis, even as modern systemic agents are integrated into clinical practice.

It is well established that PCI can induce objectively measurable neurotoxic effects, as supported by pooled analyses [15,32,33,34,35,36,37]. However, clinical relevance and persistence of these effects require scrutiny. Our synthesis substantiates the therapeutic rationale for PCI by revealing that such toxicities are predominantly limited in scope and follow a transient temporal profile. For instance, while clinician-reported common terminology criteria for adverse events (CTCAE) grade 1–2 memory impairment and cognitive disturbance were significantly higher with PCI than with observation, patient-reported outcomes from the same cohort showed comparable prevalence of self-reported memory impairment in both groups [36]. However, we acknowledge that global screenings, such as the Mini-Mental State Examination, may miss subtle deficits. To further optimize the risk–benefit ratio, strategies such as hippocampal-avoidance PCI and the administration of neuroprotective agents like memantine are essential. By sparing the site of neurogenesis, hippocampal-avoidance PCI aims to mitigate cognitive sequelae without compromising intracranial control, facilitating a more personalized, risk-adapted approach.

The present meta-analysis indicates that PCI may offer a survival benefit for LS-SCLC patients staged with MRI, yet several methodological constraints require consideration. The dependence on retrospective data introduces inherent risks regarding the precision and uniformity of reporting across different institutions. While the aggregate analysis reached statistical significance, individual cohorts occasionally yielded nonsignificant results. These discrepancies likely stem from underpowered sample sizes or varying degrees of surveillance rigor among the original study populations. The significant heterogeneity observed reflects the unavoidable diversity in clinical practice, particularly regarding PCI dose fractionation and the frequency of MRI follow-up. Although subgroup analyses helped clarify some sources of variability, the lack of standardized protocols across centers remains a potential source of bias in the final estimation of treatment effect.

A lack of granular data on salvage treatments for BM presents an additional confounding variable. The effectiveness of these subsequent interventions could influence OS and potentially cloud the direct impact attributed to PCI. Furthermore, while formal tests showed no publication bias, their statistical power is limited by the sample size of eleven studies, meaning a nonsignificant result does not definitively exclude the possibility of selective reporting. Regarding the exclusion of non-English publications, this decision was driven by the necessity for absolute accuracy in extracting complex clinical endpoints such as neurocognitive scores. This approach is unlikely to compromise the integrity of the synthesis because most of the high-impact research from leading oncology centers in East Asia is now routinely disseminated in English for global visibility. These limitations reflect the current landscape of real-world evidence and suggest that while our conclusions are robust, clinical application requires a risk-adapted approach for individual patients.

Moving forward, future research should prioritize large-scale, prospective randomized trials to directly compare PCI against standardized, high-frequency MRI surveillance in the LS-SCLC population. Identifying molecular or radiological biomarkers that can predict brain metastasis risk will also be crucial for moving toward a truly risk-adapted approach. Ultimately, in an era of enhanced systemic control and high-resolution imaging, the role of PCI should be defined not as a universal mandate but as a personalized intervention that balances consolidative efficacy with long-term neurocognitive safety.

## 5. Conclusions

In conclusion, our meta-analysis suggests that PCI may confer a survival benefit for patients with LS-SCLC who undergo brain MRI assessment after initial treatment, regardless of their subsequent MRI surveillance status. The associated neurocognitive risks appear largely limited and transient, supporting a favorable risk–benefit profile and underscoring the clinical utility of PCI in this population. These findings support the continued use of PCI in the modern MRI era, while emphasizing the need for future prospective studies to refine patient selection through biomarkers and hippocampal-avoidance techniques to maximize net clinical benefit.

## Figures and Tables

**Table 1:** General characteristics of included studies in the meta-analysis.

Author (Year)	Total Patients (PCI/Non-PCI)	Median Age, Years	Gender (Male/Female)	Smoking Status (Yes/No/Unknown)	TNM Stage (I–II/III)	Previous Treatment (CCRT/SCRT)	Treatment Response (CR/PR)	PCI Dose Gy/F	Brain MRI Time Points	Outcomes
Zhu et al. (2014) [16]	193 (67/126)	56	150/43	105/88/0	92/101	/	/	25/10	Before PCI	OS, BM rate, BMFS
Eze et al. (2017) [25]	184 (71/113)	63	111/73	/	/	68/115	/	30/15	Before PCI	OS, PFS, BM rate, BMFS
Mamesaya et al. (2018) [26]	38 (19/19)	63	23/15	36/2	10/28	28/10	13/25	25/10	Before PCI	OS; PFS
Pezzi et al. (2020) [27]	168 (84/84)	62	96/72	106/24/38	/	/	62/32	25–30/10–15	Before PCI	OS
Inoue et al. (2021) [28]	64 (32/32)	/	53/11	/	/	/	/	24–30/10–15	Before PCI; MRI surveillance	OS; BMFS
Li et al. (2021) [18]	138 (69/69)	/	101/37	84/54/0	/	60/78	50/88	25/10	Before PCI; MRI surveillance	OS, BM rate, BMFS
Qi et al. (2022) [21]	150 (75/75)	/	112/38	/	48/102	64/106	24/126	/	Before PCI; MRI surveillance	OS; BM rate
Chen et al. (2022) [29]	648 (324/324)	57	461/187	277/371/0	116/532	247/401	190/458	25–30/10	Before PCI; MRI surveillance	OS, PFS, BM rate
Pan et al. (2023) [11]	116 (83/33)	58	92/24	/	8/108	82/34	53/63	25/10	Before PCI	OS, PFS, BMFS PFS
Ito et al. (2024) [19]	75 (25/50)	/	53/22	/	11/64	51/24	75/0	25–30/10–15	Before PCI; MRI surveillance	OS; PFS
Yao et al. (2024) [30]	120 (65/55)	/	83/37	72/48/0	26/94	27/93	26/94	25/10	Before PCI; MRI surveillance	OS

Abbreviation: PCI: prophylactic cranial irradiation; TNM: Tumor-Node-Metastasis; CCRT: concurrent chemoradiotherapy; SCRT: sequential chemoradiotherapy; CR: complete response; PR: partial response; MRI: magnetic resonance imaging; OS: overall survival; BM: brain metastases; BMFS: brain metastasis-free survival; PFS: progression-free survival.

**Table 2:** Neurocognitive and quality of life outcomes of PCI: a summary from randomized controlled trials.

Study (First Author, Year)	NCF Assessment Tools	QoL and Symptom Assessment Tools	Key Findings
Gregor et al. (1997) [32]	PASAT, CFT, AVLT (learning and retention)	RSCL; HADS	NCF: Substantial and similar rates of baseline cognitive impairment in both arms (e.g., PASAT impairment: PCI 24% vs. Control 24%). No consistent evidence of major impairment attributable to PCI during follow-up. QoL: Greater symptom deterioration (e.g., tiredness, lack of energy) from baseline to 6 months in the control arm. No differences in anxiety/depression.
Slotman et al. (2008) [33]	EORTC QLQ-C30 (cognitive functioning scale)	EORTC QLQ-C30; QLQ-BN20	NCF: A trend for greater decline in patient-reported cognitive functioning with PCI (not significant). QoL: PCI had a significant short-term negative impact on fatigue and hair loss. Impact on global health status, role, emotional, and cognitive functioning was limited, with the largest mean difference (cognitive functioning at 3 months: 8.8 points) below the 10-point threshold for clinical significance.
Sun et al. (2010) [34]	MMSE, HVLT, ADLS	EORTC QLQ-C30; QLQ-BN20	NCF: No significant differences in MMSE (global cognition) or ADLS. HVLT showed a significant decline in immediate and delayed recall in the PCI arm at 1 year. QoL: No statistically significant differences in any QoL domain at 1 year.
Canney et al. (2015) [35]	ACE-R	EORTC QLQ-C30/BN20; HADS	NCF: No statistically significant difference in ACE-R scores between arms. QoL/Mood: No significant differences in global QoL, symptom scales, anxiety, or depression.
Takahashi et al. (2017) [15]	MMSE	N/A (adverse events recorded via CTCAE)	NCF: No significant differences in MMSE scores between the PCI and observation groups at baseline, 12 months, or 24 months. Toxicity: PCI was associated with higher rates of early adverse events (e.g., anorexia and malaise).
De Ruysscher et al. (2018) [36]	CTCAE v3 (Physician-assessed: memory impairment and cognitive disturbance)	EORTC QLQ-C30/BN20; EuroQol 5D	NCF: Physician-reported Grade 1–2 memory impairment (30% vs. 8%) and cognitive disturbance (19% vs. 3%) were significantly increased in the PCI arm. Patient-reported memory impairment was common in both arms (57% vs. 54%) and not significantly different. QoL: QoL was worse only at 3 months post-PCI, then comparable to observations, with no significant long-term difference (up to 48 months).
Maldonado et al. (2021) [37]	MMSE	EORTC QLQ-C30; QLQ-LC13	NCF: No significant differences in MMSE scores between arms at baseline, 3, 6, 9, and 12 months. QoL: Global health status/QoL scores improved over time in both arms, with no significant inter-arm difference in the change from baseline at 12 months.

Abbreviations: PCI: prophylactic cranial irradiation; NCF: neurocognitive function; QoL: quality of life; PASAT: Paced Auditory Serial Addition Test; CFT: Rey–Osterrieth Complex Figure Test; AVLT: Auditory Verbal Learning Test; RSCL: Rotterdam Symptom Checklist; HADS: Hospital Anxiety and Depression Scale; EORTC QLQ: European Organization for Research and Treatment of Cancer Quality of Life Questionnaire; MMSE: Mini-Mental State Examination; HVLT: Hopkins Verbal Learning Test; ADLS: Activities of Daily Living Scale; ACE-R: Addenbrooke’s Cognitive Examination-Revised; CTCAE: Common Terminology Criteria for Adverse Events.

**Table 3:** Quality assessment for the included studies using the Newcastle–Ottawa Scale.

Study	Selection	Comparability	Outcome			Total Score
			Assessment of Outcome	Follow-Up Long Enough for Outcomes	Adequacy of Follow-Up of Cohorts	
Zhu et al. (2014) [16]	4	1	1	1	1	8
Eze et al. (2017) [25]	3	1	1	1	1	7
Mamesaya et al. (2018) [26]	3	2	1	1	0	7
Pezzi et al. (2020) [27]	4	2	1	1	1	9
Inoue et al. (2021) [28]	3	2	1	1	0	7
Li et al. (2021) [18]	3	2	1	0	1	7
Qi et al. (2022) [21]	3	2	1	1	0	7
Chen et al. (2022) [29]	3	2	1	1	1	8
Pan et al. (2023) [11]	3	2	1	1	1	8
Ito et al. (2024) [19]	3	2	1	1	1	8
Yao et al. (2024) [30]	2	2	1	1	1	7

Note: NOS scores range from 0 to 9; scores ≥ 7 indicate high quality.

## Data Availability

The datasets used and/or analyzed during the current study are available from the corresponding author on reasonable request.

## Supplementary Materials

The following supporting information can be downloaded at https://www.mdpi.com/article/10.3390/cancers18121970/s1. Figure S1. Bubble plots for univariable meta-regression of potential moderators for overall survival (OS). (a) Meta-regression on publication year (P = 0.108); (b) Meta-regression on complete response (CR) rate (P = 0.236); Table S1. PRISMA Checklist.
